# Case report: Detection of fetal trisomy 9 mosaicism by multiple genetic testing methods: Report of two cases

**DOI:** 10.3389/fgene.2023.1121121

**Published:** 2023-03-10

**Authors:** Na Ma, Zhenhua Zhu, Jiancheng Hu, Jialun Pang, Shuting Yang, Jing Liu, Jing Chen, Wanglan Tang, Haiyan Kuang, Rong Hu, Zhuo Li, Hua Wang, Ying Peng, Hui Xi

**Affiliations:** ^1^ Department of Medical Genetics, Hunan Provincial Maternal and Child Healthcare Hospital, Changsha, China; ^2^ Department of General Surgery, Changsha Central Hospital Affiliated to University of South China, Changsha, China; ^3^ Department of Ultrasonography, Hunan Provincial Maternal and Child Healthcare Hospital, Changsha, China; ^4^ Center for Medical Genetics and Hunan Key Laboratory of Medical Genetics School of Life Sciences, School of Life Sciences, Central South University, Changsha, China; ^5^ Department of Medical Genetics, Hunan Children’s Hospital, Changsha, China

**Keywords:** karyotype, copy number variation sequencing, non-invasive prenatal testing, chromosomal microarray analysis, mosaicism, trisomy 9

## Abstract

Chromosomal mosaicism remains a perpetual diagnostic and clinical dilemma. In the present study, we detected two prenatal trisomy 9 mosaic syndrome cases by using multiple genetic testing methods. The non-invasive prenatal testing (NIPT) results suggested trisomy 9 in two fetuses. Karyotype analysis of amniocytes showed a high level (42%–50%) of mosaicism, and chromosomal microarray analysis (CMA) of uncultured amniocytes showed no copy number variation (CNV) except for large fragment loss of heterozygosity. Ultrasound findings were unmarkable except for small for gestational age. In Case 1, further umbilical blood puncture confirmed 22.4% and 34% trisomy 9 mosaicism by CMA and fluorescent *in situ* hybridization (FISH) respectively. After comprehensive consideration of the genetic and ultrasound results, the two gravidas decided to receive elective termination and molecular investigations of multiple tissue samples from the aborted fetus and the placenta. The results confirmed the presence of true fetoplacental mosaicism with levels of trisomy 9 mosaicism from 76% to normal in various tissues. These two cases highlight the necessity of genetic counseling for gravidas whose NIPT results highly suggest the risk of chromosome 9 to ascertain the occurrence of mosaicism. In addition, the comprehensive use of multiple genetic techniques and biological samples is recommended for prenatal diagnosis to avoid false-negative results. It should also be noted that ultrasound results of organs with true trisomy 9 mosaicism can be free of structural abnormalities during pregnancy.

## Introduction

Trisomy 9 is an uncommon chromosomal abnormality that can occur in a mosaic or non-mosaic state (Cantú et al., 1996). Full trisomy 9 syndrome can be lethal with an incidence of 2.2%–2.7% in first-trimester spontaneous abortions (Ferreres et al., 2008; Benn and Grati., 2021), but trisomy 9 mosaicism syndrome has been reported compatible with life (Bruns and Campbell. 2015). More than 100 cases of mosaic trisomy 9 have been reported in the literature (Li et al., 2021). Most individuals with trisomy 9 and trisomy 9 mosaicism have prenatal and perinatal issues, including intrauterine growth retardation or “small size”, oligohydramnios, placental insufficiency, premature rupture of membranes, and skeletal abnormalities (Chen et al., 2010; Bruns and Campbell. 2015). Although there is no specific intrauterine ultrasound phenotype, more uncommon mosaic chromosomal abnormalities have been detected prenatally by invasive genetic tests following the sonographic diagnosis of fetal anomalies or high risk of the non-invasive prenatal testing (NIPT) (Wang et al., 2020; Lee et al., 2018). The diagnosis of chromosomal mosaicism in the prenatal stage is fraught with uncertainty and multiple factors need to be considered in order to gauge the likely impact. In genetic counseling for mosaicism, multiple influencing factors need to be considered comprehensively, including the chromosome position, type of mosaicism, distribution of the abnormal cell line in the fetus, assay noise, and culture artifacts. Herein, we present two cases of fetoplacental mosaic trisomy 9 based on a series of results from prenatal screening to diagnosis and follow-up observations on abnormal cell distributions in various tissues within the fetus. It should be addressed that ultrasound results of organs with true trisomy 9 mosaicism can be free of structural abnormalities during pregnancy.

## Case report

### Case 1

The gravida was a 26-year-old woman (gravida 2, para 0) with no family history of chromosomal abnormalities and a sign of spontaneous abortion during early pregnancy. She underwent maternal serum screening at 12 weeks of gestation, which revealed a risk for Down syndrome of 1 in 200. Due to concerns about the unavoidable risks of invasive prenatal diagnosis methods, she chose NIPT for fetal autosomal aneuploidies screening at 16^+1^ weeks of gestation after genetic counseling. Sample preparation, maternal plasma DNA sequencing, and bioinformatics analysis for NIPT were carried out using BGI platform (MGISEQ-2000, Shenzhen, China) as previously described (Xiang et al., 2023). NIPT analysis showed that the Z-scores of other chromosomes were all in the normal range (−3 < Z < 3) except for chromosome 9 (Z = 8.9898) with a cell-free fetal DNA fraction at 11.08% (3.5% is the least reliable cell-free fetal DNA level), suggesting a possibility of trisomy 9 or trisomy 9 mosaicism.

**FIGURE 1:**
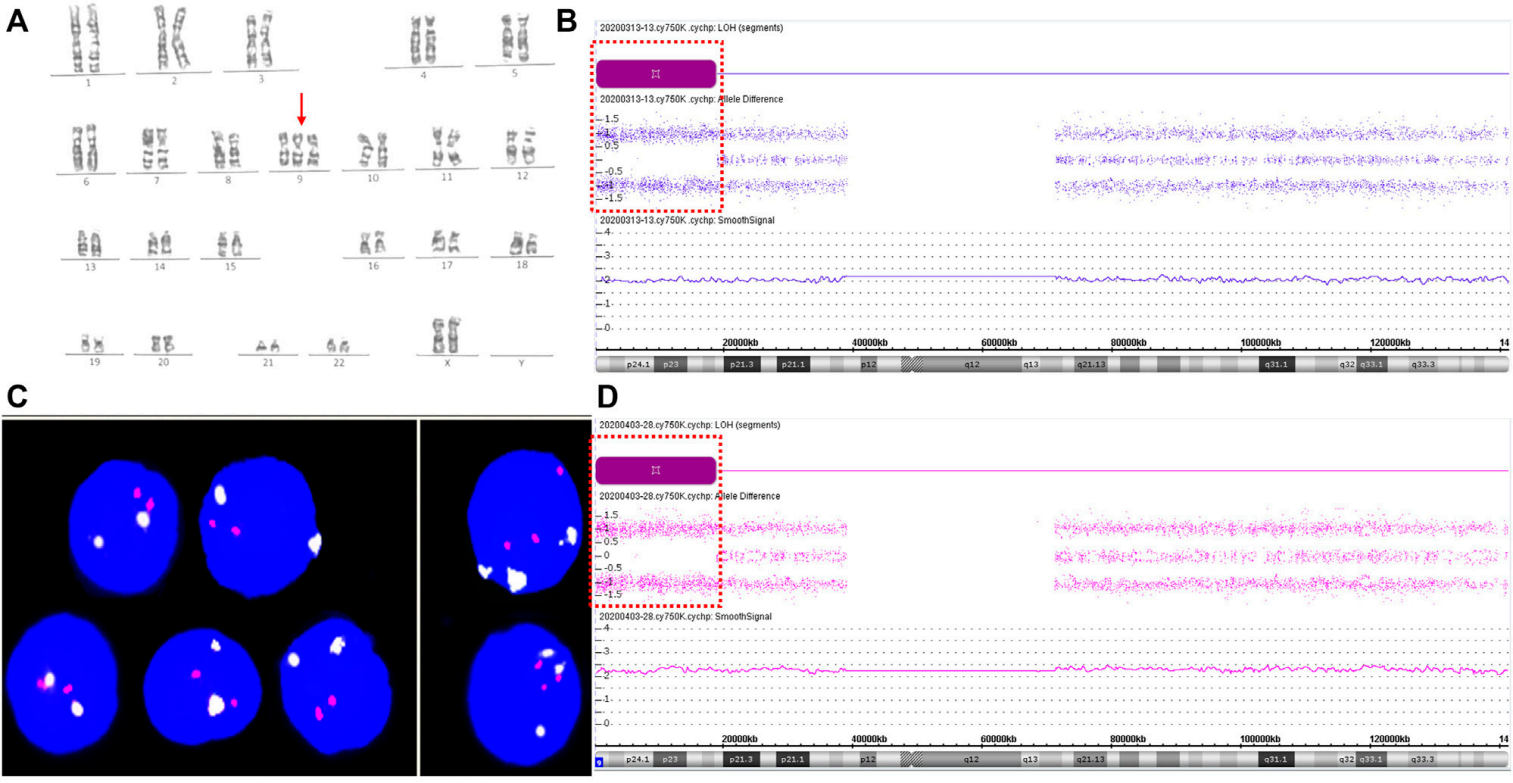
Results for trisomy 9 mosaicism in Case1. **(A)** Karyotype profiles indicating trisomy 9 (red arrows indicate the chromosome 9). **(B)** CMA assay results of the uncultured amniotic fluid sample for Case 1. Loss-of-heterozygosity (LOH) at 9p24.3p22.1 detected by CMA (positions of LOH are indicated by the dashed boxes). **(C)** FISH profiles of 22.4% trisomy 9 mosaicism in uncultured interphase umbilical blood cells using chromosome 9 centromere-specific (white) and 9qter telomeres-specific probe (red). **(D)** CMA assay results of uncultured umbilical blood sample for Case 1. LOH at 9p24.3p22.1 detected by CMA (positions of LOH are indicated by the dashed boxes). SmoothSignal showed 34% trisomy 9 mosaicism (*x*-axis: chromosomes 9; *y*-axis: copy number).

Following post-test genetic counseling for the NIPT results, the gravida agreed to receive amniocentesis for further analysis at 22 weeks of gestation. Genetic amniocentesis test revealed a karyotype of mos 47,XX,+9 [25]/46,XX [25], indicating a level of 50% trisomy 9 mosaicism (Figure 1A). Parental karyotypes of peripheral blood were normal. Chromosomal microarray analysis (CMA) of uncultured amniocytes with the Affymetrix CytoScan^®^750 K Array (Affymetrix Inc., CA, United States) revealed an 18.54 Mb loss of heterozygosity at 9p24.3p22.1, arr [GRCh37] 9p24.3p22.1 (216,123_18,758,836) × 2 hmz (Figure 1B). As there was no copy number variation (CNV) in the CMA results, and the prenatal ultrasound findings at 23^+3^ weeks of gestation were unremarkable, further invasive prenatal diagnosis by percutaneous umbilical blood sampling was performed at 27 weeks of gestation to confirm the trisomy 9 mosaicism. Karyotype analysis of umbilical blood identified only 1 cell with an abnormal 47,XX,+9 karyotype among 100 metaphase cells. According to the International System for Human Cytogenomic Nomenclature (McGowan-Jordanet al., 2020) guidelines and recommendations (McGowan-Jordanet al., 2020), this karyotype should be normally reported as 46,XX. Interphase fluorescent in situ hybridization (FISH) analysis on uncultured fetal cord blood showed 22.4% (35/156) trisomy 9 mosaicism (Figure 1C). CMA analysis on the DNA extracted from uncultured umbilical blood detected a gene dosage increase of chromosome 9 and loss of heterozygosity for a large fragment of chromosome 9: arr [GRCh37] 9p24.3q34.3 (208,454_141,018,648) × 3 [0.34]; arr [GRCh37] 9p24.3p22.1 (216,123_18,782,021) × 2 hmz (Figure 1D). Ultrasound findings at 28^+4^ weeks of gestation showed no structural abnormalities except for those slightly smaller than the gestational age, with a biparietal diameter of 7.8 cm (97.5th), a head circumference of 26.1 cm (14.7th), an abdominal circumference of 21.8 cm (2nd), a humerus length of 4.7 cm (14.7th), a femur length of 5.2 cm (14.7th), and a fetal weight assessment of 1040 g (5.5th). The gravida chose to terminate the pregnancy at 29^+4^ weeks of gestation.

A subsequent autopsy of the aborted fetus confirmed the prenatal ultrasound finding of no structural anomalies. The copy number variation sequencing (CNV-seq) (NextSeq CN500 platform, Berry Genomics, Beijing, China) analysis of the maternal and fetal center of the placenta verified trisomy 9 with the level of 80% and 81% mosaicism respectively. The levels of trisomy 9 mosaicism were 6% in the DNA of uncultured amniocytes following CMA testing, 23% in uncultured umbilical blood, and 21% in the umbilical cord (Supplementary Figure S1). The trisomy 9 mosaicism levels within various tissues were 60% in the uterus, 35% in the lung, 20% in the kidney, 15% in the skeletal muscle, 15% in the small intestine, 11% in the ovaries, and 10% in the heart. No trisomy 9 mosaicism was detected in the skin, thymus, adrenal glands, and brain (Supplementary Figure S2).

### Case 2

**FIGURE 2:**
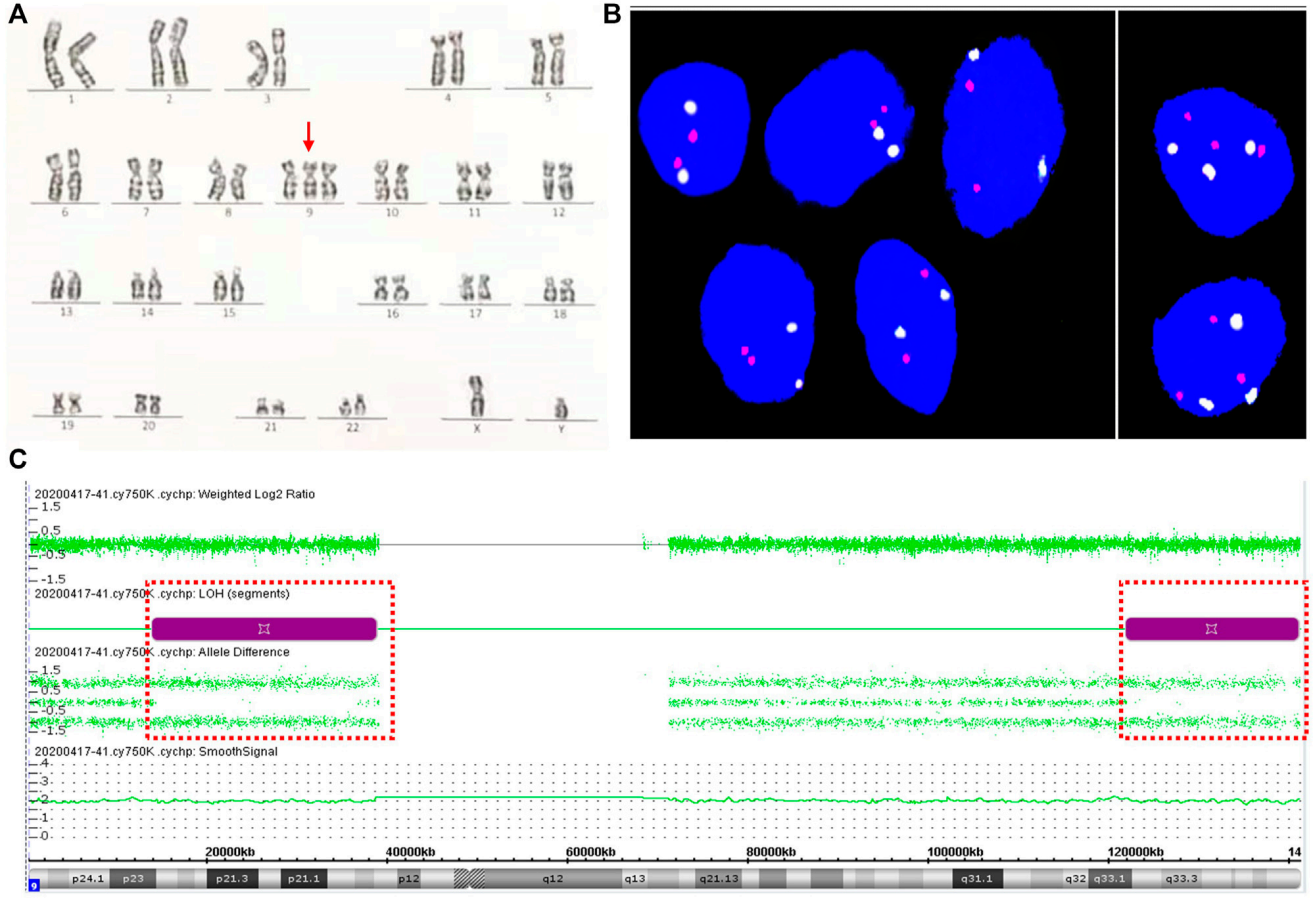
Results for trisomy 9 mosaicism in Case 2. **(A)** Karyotype profiles indicating trisomy 9 (red arrows indicated the chromosome 9). **(B)** FISH profiles of 8.2% trisomy 9 mosaicism in interphase uncultured oral mucosal cells using chromosome 9 centromere-specific (white) and 9qter telomeres-specific probe (red). **(C)** CMA assay results of uncultured amniocytes for Case 2. LOH at 9p23p13.1 and 9q33.1q34.3 detected by CMA (positions of LOH are indicated by the dashed boxes).

A 31-year-old woman (gravid 2, para 0) was referred to the Hunan Provincial Maternal and Child Health Care Hospital because of the high risk of NIPT results. NIPT using the MGISEQ-2000 platform (BGI, Shenzhen, China) at 14 gestational weeks showed that the Z-score of chromosome 9 was outside the normal range (14.8715, 11.931% cell-free fetal DNA fraction), suggesting a high risk of trisomy 9. There was no signs of miscarriage during early pregnancy, and all common laboratory parameters were within normal reference ranges. The gravida underwent amniocentesis at 18 weeks of gestation. Genetic amniocentesis analysis revealed the existence of trisomy 9 mosaicism, mos 47,XY,+9 [21]/46,XY [29] with a 42% level of trisomy 9 mosaicism (Figure 2A). Parental karyotypes of peripheral blood were normal. CMA of uncultured amniocytes with the Affymetrix CytoScan^®^750 K Array showed no CNV on chromosome 9 except for 24.96 Mb and 19.22 Mb loss of heterozygosity at 9p23p13.1 and 9q33.1q34.3: arr [GRCh37] 9p23p13.1 (13,814,169_38,771,831) × 2 hmz and arr [GRCh37] 9q33.1q34.3 (121,790,571_141,011,581) × 2 hmz (Figure 2B). Prenatal ultrasound at 20^+5^ weeks of gestation showed no structural anomalies except for a slightly wider bilateral renal pelvis (0.5–0.52 cm for the left side and 0.6–0.63 cm for the right side). The pregnancy was terminated at 21^+5^ weeks of gestation upon the request of the parents.

Upon approval from the parents, an autopsy of the aborted fetus was performed, and the result showed no significant structural anomalies. FISH analysis of uncultured oral mucosal cells with chromosome 9p-ter and 9q-ter FISH probes detected trisomy 9 in 16 (8.2%) of 196 cells examined (Figure 2C). CNV-seq analysis of the maternal and fetal center of the placenta confirmed placental mosaicism with chromosome 9 of 2.76 triploid equivalents (Supplementary Figure S3). CNV-seq of the multiple tissue samples of the aborted fetus confirmed true fetal mosaicism. The levels of trisomy 9 mosaicism within various tissues were variable: 10% in the cord portion close to the fetus, 15% in the cord portion close to the placenta (Supplementary Figure S3), 20% in the heart, 12% in the skeletal muscle, 10% in the brain, 8% in the stomach, and 6% in the adrenal glands. Trisomy 9 was not detected in cutaneous cells, small intestine, kidney, and the DNA of uncultured amniocytes following CMA testing (Supplementary Figure S4).

## Discussion

Owing to the advantage of NIPT in detecting genome-wide chromosomal anomalies (Bianchi and Wilkins-Haug., 2014), more studies have investigated the use of NIPT in detecting rare autosomal trisomies, including trisomy 9 (Lee et al., 2018; Wang et al., 2020; Li et al., 2022). However the positive predictive value of NIPT for rare autosomal trisomies is low (4%–6%) in the general obstetrical population (van der Meij et al., 2019; Van Den Bogaert et al., 2021), and approximately 40% of all rare autosomal trisomy cases culminate in adverse perinatal outcomes (Xiang et al., 2023). Since most of the circulating fetal DNA in maternal plasma is derived primarily from the placental trophoblasts (Flori et al., 2004), and trisomy 9 is usually miscarried in the first trimester (López-Félix et al., 2017), invasive prenatal testing is strongly recommended for all gravidas with positive NIPT results of trisomy 9 to exclude placental or fetal mosaicism.

Different genetic techniques have respective advantages in detecting mosaicism, and attention should be paid to excluding culture artifacts in detecting the mosaicism of cultured samples. For the flask method of karyotyping, we followed the current international practice and analyzed at least 50 cells from a minimum of two different culture vessels. Cytogenetic abnormalities confined to two or more flasks are considered level III true mosaicism (Hsu and Benn., 1999). Nevertheless, with the developments in molecular genetics, more studies have demonstrated inconsistent mosaic ratios between the results of cultured karyotype and uncultured CMA or FISH (Chen et al., 2016, Chen et al., 2012, Ma et al., 2015). Inconsistent results between CNV-seq/CMA (uncultured samples) and karyotyping (cultured samples) in the prenatal diagnosis of mosaic trisomy 9 may be attributed to the variable proliferation of cells with different karyotypes under *in vitro* cell culture, which is especially common in prenatal diagnosis of mosaic trisomy 9 (Chen et al., 2010; Tang et al., 2019). The growth advantages of trisomy 9 amniotic fluid cells is entirely useless in cord blood cell culture in our cases as previously reported (Miryounesi et al., 2016), which may be the reason why trisomy 9 is less frequently diagnosed in children by karyotyping (Li et al., 2021). Therefore, attention should be paid to culture artifacts and technical limitations of karyotyping in prenatal diagnosis of mosaicism. Besides, umbilical blood sampling for rapid confirmation of trisomy 9 mosaicism by karyotyping is not practical and extensive use of multiple techniques is strongly suggested in prenatal diagnosis of mosaicism suggested by NIPT.

The clinical significance of uniparental disomy (UPD) lies in its ability of producing either aberrant patterns of imprinting or homozygosity for recessive mutations. GLIS3 (MIM: 610199) is only paternally imprinted gene on chromosome 9, which is implicated in neonatal diabetes and pancreatic development by autosomal recessive inheritance instead of imprinting (Yang et al., 2011; Kang et al., 2009). According to the molecular results performed in the present cases, the large block(s) of homozygosity of chromosome 9 detected by SNP-array in both amniocytes and umbilical blood is most probably the result of postzygotic trisomy rescue combined with mitotic recombination. Among the 5135 cases included in ChromosOmics Database (Liehr, 2023, https://cs-tl.de/DB/CA/UPD/0-Start.html), only 53 cases have been referred to chromosome 9 and limited prenatal clinical significance of UPD 9 has been reported in the literature (Slater et al., 2000; Chen et al., 2022, Chen et al., 2010). Besides, the UPDs of most cases were reported after the discovery of trisomy 9 mosaicism at cytogenetic prenatal diagnosis (Liehr, 2023, https://cs-tl.de/DB/CA/UPD/0-Start.html), which makes the clinical phenotype analysis of UPD 9 more difficult.

Chromosomal mosaicism presents a major interpretative dilemma in prenatal genetic counseling. Genetic counseling for clinical outcomes of chromosomal mosaicism in pregnant women needs to be assessed on the case-by-case basis and comprehensively consider multiple factors, including the timing of the initial event, gene-phenotype associations of referred chromosome, the ratio and distribution of the normal/abnormal cells in tissues. The placental findings from NIPT and the trisomic rescue observed by SNP-array and karyotyping, suggest that our cases are fetoplacental trisomy 9 mosaicism, which was further confirmed by CNV-seq testing of the aborted placental and fetal tissues. Prenatal clinical features of mosaic trisomy 9 are often complicated with intrauterine growth retardation and/or “small” size, which is not specific. Live births with trisomy 9 mosaicism may present with characteristic phenotypic features, such as craniofacial abnormalities (small palpebral fissures, bulbous nose, micrognathia, abnormal ears, scoliosis, low-set ears and micrognathia), cardiac abnormalities, feeding (gastroesophageal reflux) and breathing difficulties, cryptorchidism, hip dysplasia, seizures, and developmental delay (Li et al., 2021; Bruns and Campbell. 2015). The incidence and severity of malformations and intellectual disability correlate with the percentage of trisomic cells in different tissues (Lee et al., 2018), while the types of tissue sources that can be obtained for prenatal diagnosis are limited. Furthermore, the fetus in this report exhibited no structural abnormality in prenatal ultrasound, suggesting that fetal organs with trisomy 9 mosaicism may not always present with abnormal clinical manifestations during the prenatal period. Therefore, genetic counseling for evaluating the likely phenotype of mosaic trisomy 9 in prenatal diagnosis is very difficult and challenging.

In conclusion, the findings in the present study suggest that attention should be paid to the possibility of mosaicism and placental mosaicism in gravidas with positive NIPT results of trisomy 9, and the comprehensive use of multiple genetic techniques and biological samples is strongly suggested for the diagnosis of trisomy 9 mosaicism. Genetic counseling for clinical outcomes of trisomy 9 mosaicism during pregnancy should be provided on the case-by-case basis by comprehensive consideration of the prenatal results and the fact that true fetal trisomy 9 mosaicism can be free of structural anomalies.

## Data Availability

The original contributions presented in the study are included in the article/Supplementary Material, further inquiries can be directed to the corresponding authors.

## Supplementary material

SUPPLEMENTARY FIGURE S1 CNV-seq profiles of Case 1 for multiple samplings showed various levels of trisomy 9. CNV-seq profiles are shown for uncultured amniotic fluid, maternal center of the placenta, fetal center of the placenta, umbilical cord, and umbilical blood of Case 1 with 6%, 81%, 80%, 21% and 23% trisomy 9 mosaicism, respectively. The blue line represents the mean copy number and the black box represents the centromere (x-axis: chromosomes 9; y-axis: copy number).

SUPPLEMENTARY FIGURE S2 CNV-seq profiles of Case 1 for multiple samplings showed various levels of trisomy 9. CNV-seq profiles are shown for uterus, lung, kidney, heart and skin of Case1 with 60%, 35%, 20% and 10% trisomy 9 mosaicism and normal result, respectively. The blue line represents the mean copy number and the black box represents the centromere (x-axis: chromosomes 9; y-axis: copy number).

SUPPLEMENTARY FIGURE S3 CNV-seq profiles of Case 2 for multiple samplings showed various levels of trisomy 9. CNV-seq profiles are shown for uncultured amniotic fluid, maternal center of the placenta, fetal center of the placenta, cord portion close to the placenta and cord portion close to the fetus of Case 2 with normal result, 76%, 76%, 15% and 10% trisomy 9 mosaicism, respectively. The blue line represents the mean copy number and the black box represents the centromere (x-axis: chromosomes 9; y-axis: copy number).

SUPPLEMENTARY FIGURE S4 CNV-seq profiles of Case 2 for multiple samplings showed various levels of trisomy 9. CNV-Seq profiles are shown for the heart, brain, stomach, adrenal glands, and skin of Case 2 with 20%, 10%, 8%, 6% trisomy 9 mosaicism and normal result, respectively. The blue line represents the mean copy number and the black box represents the centromere (x-axis: chromosomes 9; y-axis: copy number).
