# Supplementary figures and images for: Case report: Detection of fetal trisomy 9 mosaicism by multiple genetic testing methods: Report of two cases
Source: Front Genet. 2023 Mar 10;14:1121121. doi: 10.3389/fgene.2023.1121121 (PMC10036773; doi:10.3389/fgene.2023.1121121)

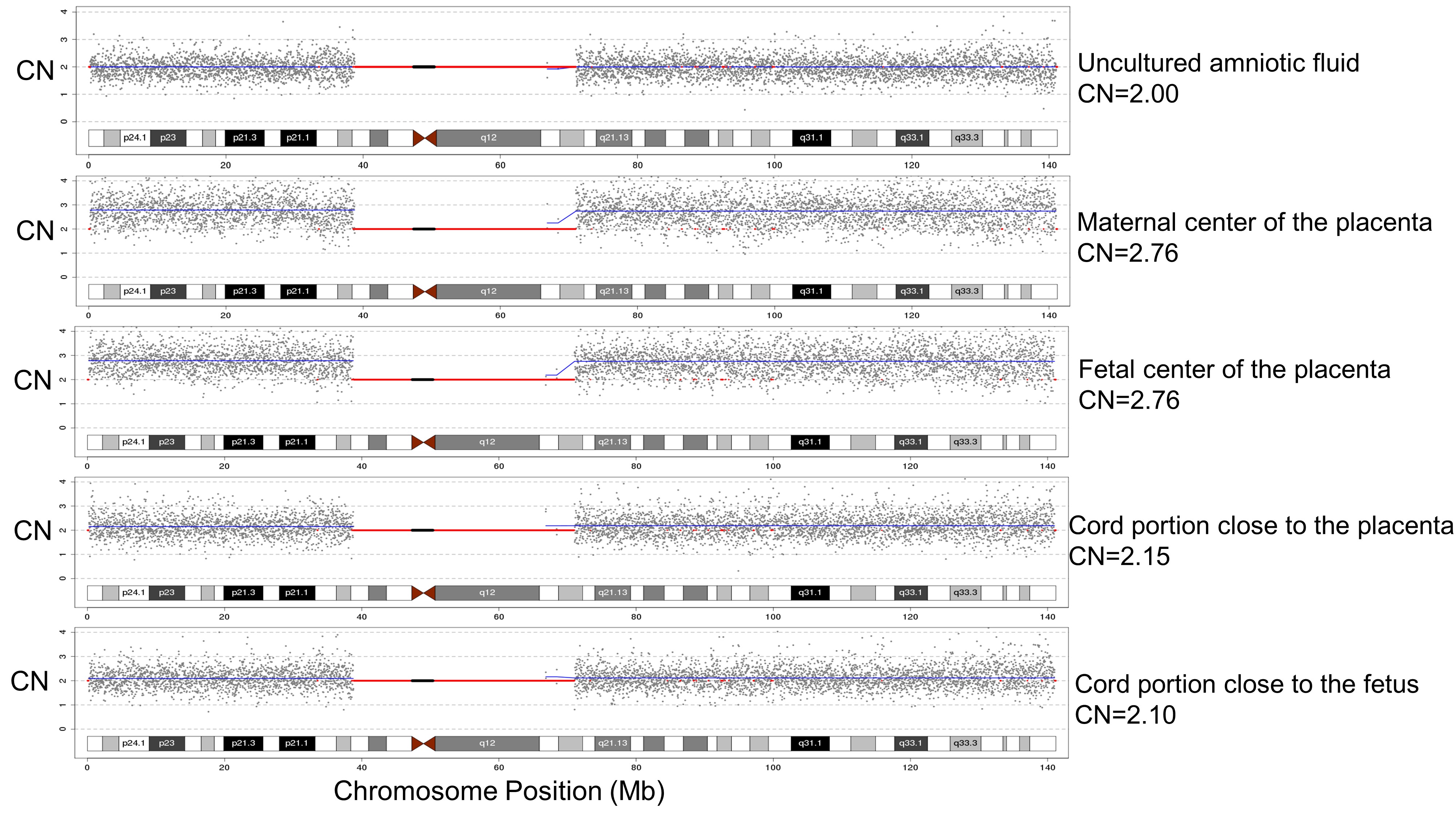

Supplement: Supplementary file 1 [file Image3.JPEG]

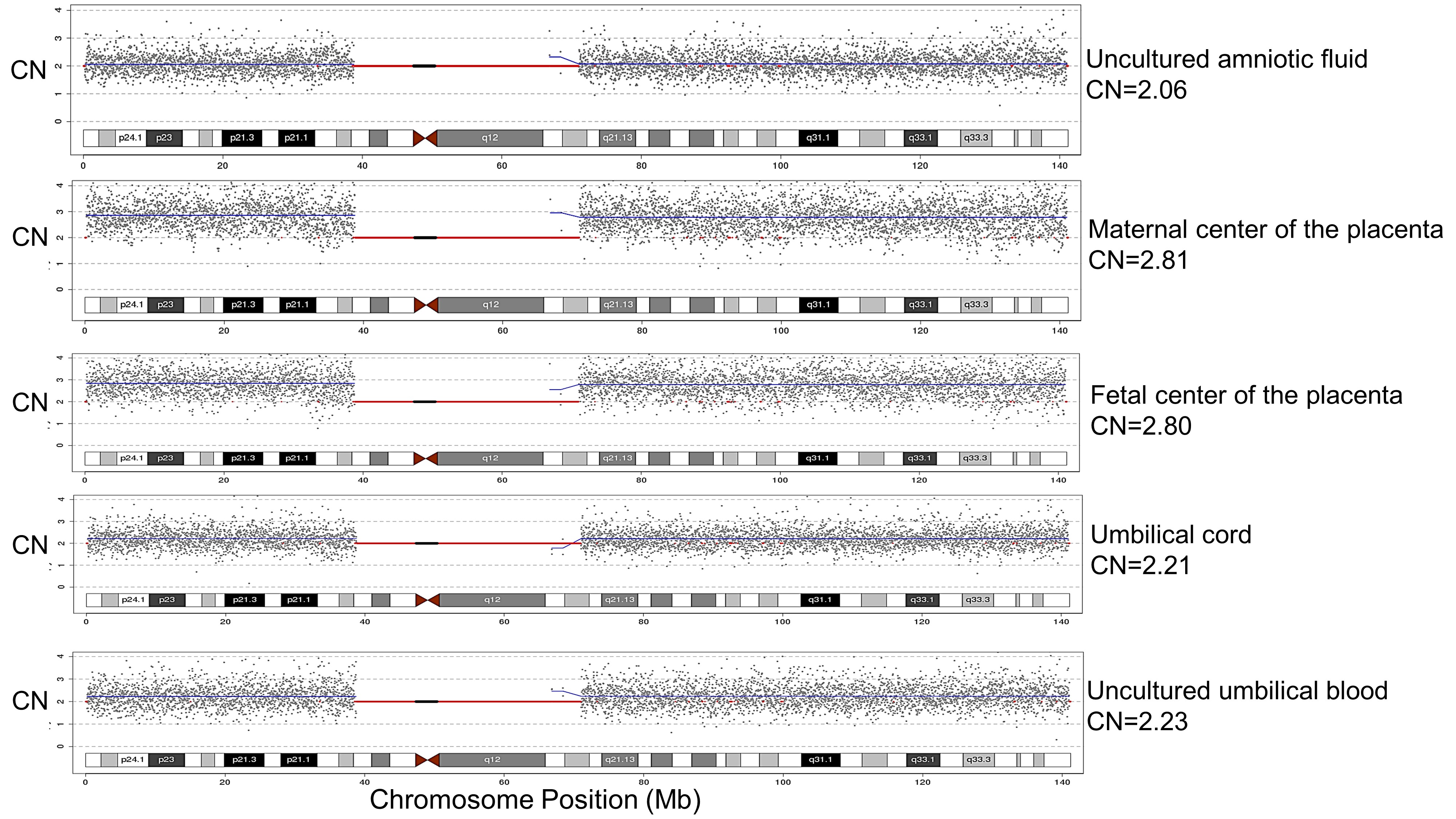

Supplement: Supplementary file 2 [file Image1.JPEG]

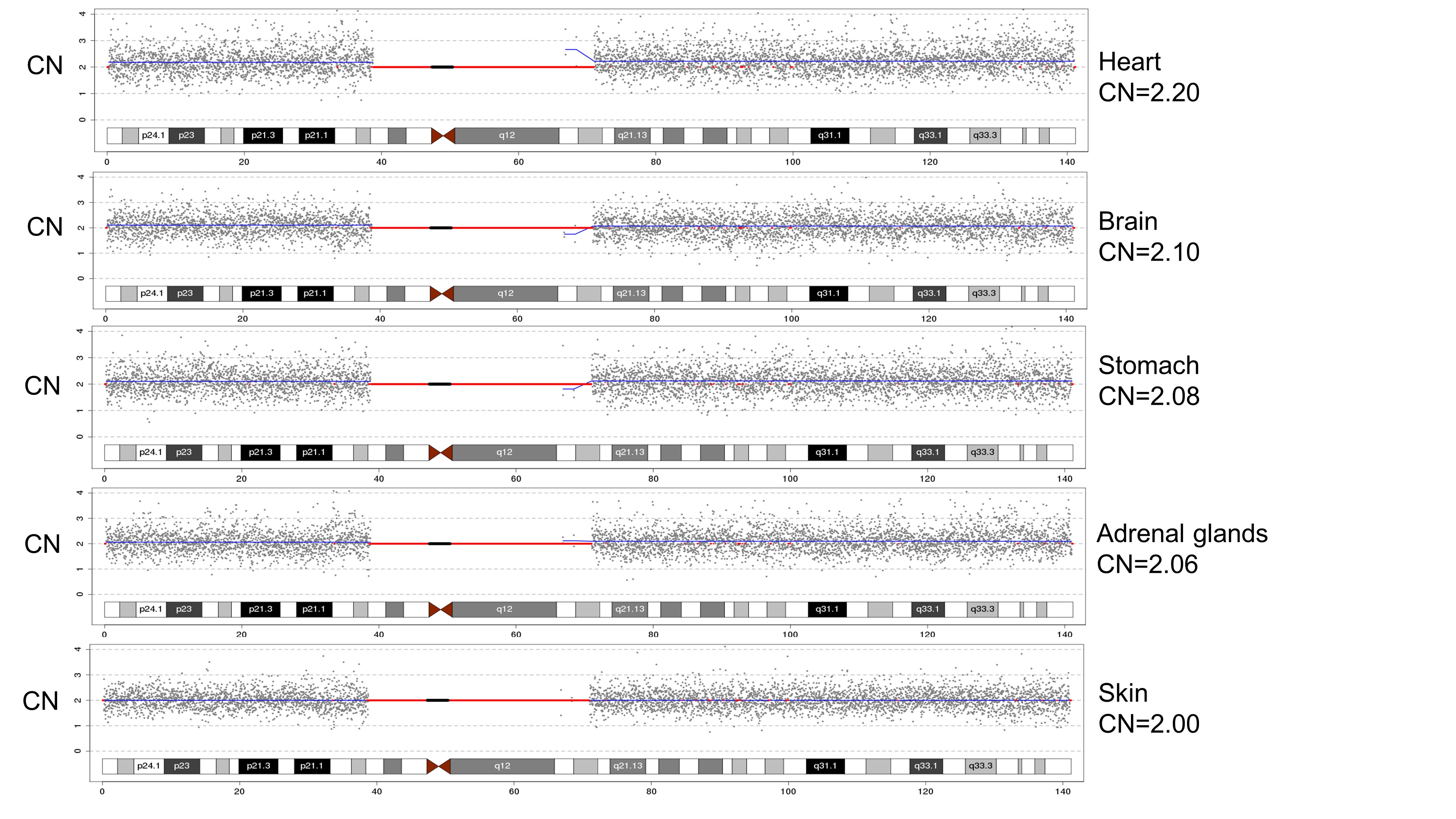

Supplement: Supplementary file 3 [file Image4.JPEG]

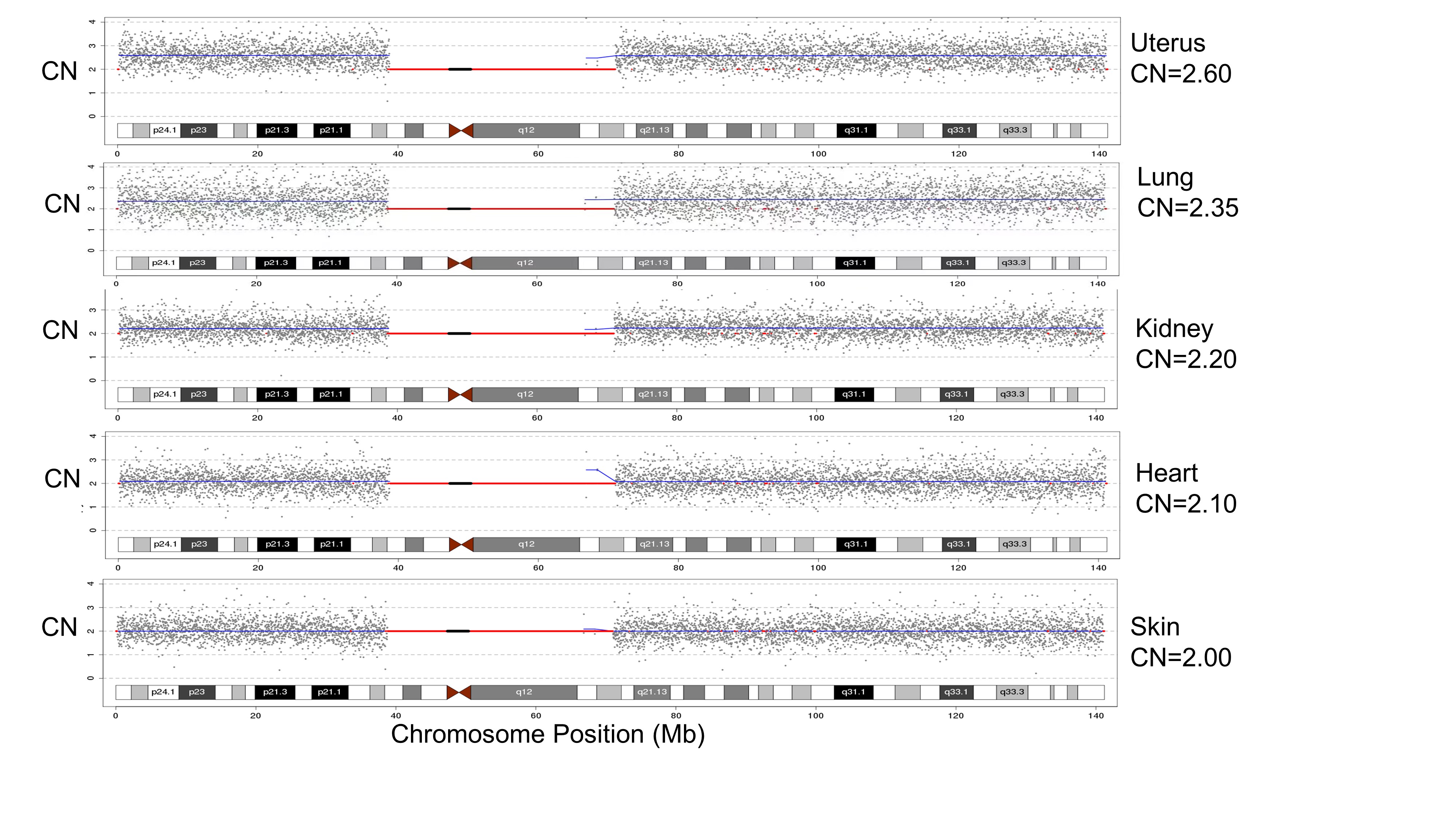

Supplement: Supplementary file 4 [file Image2.JPEG]
